# Molecular cartilage pathology following anterior cruciate ligament reconstruction: a systematic review of MRI studies

**DOI:** 10.3389/fsurg.2026.1714750

**Published:** 2026-02-24

**Authors:** Abdulmajeed Alfayyadh

**Affiliations:** Department of Physical Therapy and Health Rehabilitation, College of Applied Medical Sciences, Jouf University, Sakaka, Saudi Arabia

**Keywords:** ACL reconstruction, compositional MRI, post-traumatic osteoarthritis, quantitative MRI, T1rho, T2 mapping, tibiofemoral cartilage, UTE-T2*

## Abstract

**Background:**

Anterior cruciate ligament (ACL) injury increases the risk of post-traumatic osteoarthritis. Although ACL reconstruction (ACLR) restores mechanical stability, cartilage matrix abnormalities may persist and can be detected using quantitative and compositional magnetic resonance imaging (MRI).

**Objectives:**

To synthesize MRI evidence on tibiofemoral cartilage compositional changes after ACLR, identify factors associated with worse cartilage outcomes, and summarize methodological quality and limitations of the evidence.

**Methods:**

Embase, PubMed, Web of Science, Scopus, and Cochrane Library were searched for studies published between 2000 and 2025 that evaluated tibiofemoral cartilage after ACLR using quantitative or compositional MRI. Two reviewers performed screening, data extraction, and risk-of-bias assessment. Meta-analysis was restricted to outcomes with sufficient comparability across studies; other outcomes were synthesized narratively.

**Results:**

Thirty-five studies were included. Across cohorts, ACLR knees generally demonstrated elevated cartilage T2 relaxation times and elevated T1*ρ* values compared with contralateral or healthy control knees, consistent with persistent cartilage matrix alteration. Abnormalities were most frequently reported in weight-bearing medial compartment regions and were more consistently observed at longer follow-up intervals. Meniscal pathology and altered biomechanics were repeatedly associated with less favorable compositional or structural cartilage outcomes. Evidence regarding graft type and rehabilitation strategy was limited and heterogeneous, and causal inferences could not be made. Meta-analysis was feasible only for a subset of T2 comparisons and suggested higher tibiofemoral T2 in ACLR knees, with substantial between-study heterogeneity.

**Limitations:**

Considerable variability in MRI protocols, regions of interest, and reporting limited pooling. Several studies may represent overlapping cohorts, and many analyses were underpowered.

**Conclusion:**

MRI evidence indicates that biochemical cartilage abnormalities commonly persist after ACLR, particularly in the medial tibiofemoral compartment. Standardized imaging protocols and long-term longitudinal studies linking MRI biomarkers to clinical outcomes are needed.

**Systematic Review Registration:**

https://www.crd.york.ac.uk/PROSPERO/view/CRD420251156314, PROSPERO CRD420251156314.

## What is known about this subject

Anterior cruciate ligament reconstruction (ACLR) restores knee stability, yet many patients remain at risk for post-traumatic osteoarthritis. Quantitative and compositional MRI techniques, particularly T2 and T1rho mapping and UTE-T2* approaches, can detect early cartilage matrix abnormalities before overt structural degeneration becomes apparent.

## What this study adds to existing knowledge

This systematic review synthesizing 35 studies shows that ACLR knees commonly demonstrate persistent compositional cartilage abnormalities, most consistently reflected by elevated T2 relaxation times and elevated T1rho values compared with contralateral or healthy control knees. Abnormalities are frequently localized to weight-bearing medial tibiofemoral regions and appear more consistent at longer follow-up intervals. Meniscal pathology and altered biomechanics were repeatedly associated with less favorable cartilage outcomes, whereas evidence regarding graft type and rehabilitation strategy was limited and heterogeneous, precluding causal conclusions.

## Introduction

### Background

Epidemiological studies have established that Anterior Cruciate Ligament (ACL) injuries account for a substantial proportion of knee sports injuries across the globe (1), with an incidence of 30–78 per 100,000 patients (2, 3). The financial burden of ACL injuries, including rehabilitation and surgery, is enormous, and thus it is important to apply effective treatment strategies. ACL injuries account for the most common type of knee injury, particularly among sportsmen competing in high-level sports like soccer, basketball, and alpine skiing (4). The injuries are prone to severe functional deficits, including knee instability, loss of range of motion, and susceptibility to further injury, such as meniscal tear and post-traumatic osteoarthritis (OA) (5). Due to these challenges, anterior cruciate ligament reconstruction (ACLR) has been the gold standard therapy for patients wanting to return to high-level physical activity. Nevertheless, increased levels of physical activity after ACLR have been associated with elevated cartilage degeneration, as reflected by T2 relaxation times. These findings emphasize the significance of individualized rehabilitation protocols in the prevention of long-term degeneration of cartilage (6). Nevertheless, despite its popularity and effectiveness in restoration of knee stability, uncertainty remains about the long-term implication of ACLR on joint health, particularly on the biochemical characteristics of articular cartilage (7).

Articular cartilage is an extraordinary type of connective tissue that covers the articulating surface of bones in synovial joints to create a smooth, low-friction surface required for motion and the transmission of mechanical loads (8). Evolution of imaging methods for cartilage has improved our understanding of molecular changes related to joint degeneration after ACLR. For instance, delayed gadolinium-enhanced MRI of cartilage (dGEMRIC) has been used to evaluate changes in glycosaminoglycan (GAG) content, which is an early marker of progression of osteoarthritis (9, 10). Cartilage's extraordinary extracellular matrix, which consists mainly of collagen fibers, proteoglycans, and water, supports its mechanical properties and overall function. Biochemical modification of cartilage composition, e.g., changes in collagen fiber orientation (quantitated by T2 relaxation times) or changes in proteoglycan content (quantitated with T1rho or GAG mapping), are early markers of cartilage degeneration as well as possible precursors of OA (2). Such modifications most often become apparent prior to structural changes detectable by conventional imaging modalities, thereby illustrating the role of biochemical evaluation as an important tool for early diagnosis and treatment (11).

Magnetic resonance imaging (MRI) has proven an effective noninvasive imaging technology for evaluating cartilage biochemical makeup. Sophisticated MRI modalities, including T1rho mapping, T2 mapping, and UTE based techniques, enable noninvasive assessment of cartilage matrix status (12, 13). T2 mapping, for example, is utilized to depict collagen structure and water content, whereas T1rho mapping is commonly interpreted as sensitive to proteoglycan related matrix alteration (14). These techniques have been extensively utilized to study changes in cartilage in different patient populations, i.e., those with ACL injuries and ACLR patients (15).

### Rationale for the review

In spite of ACLR's success in restoring knee stability, increasing evidence suggests that the procedure might not restore normal knee biomechanics. Altered loading patterns and residual joint laxity after ACLR have been quoted as explanations for early cartilage degeneration (16). A number of studies have reported increased T2 relaxation times and other compositional abnormalities consistent with early cartilage matrix alteration in ACL-R cartilage, indicative of early degenerative changes (17, 18). Graft choice has been explored as a potential modifier of cartilage outcomes after ACLR, but available comparisons are limited and observational, and confounding by patient and concomitant injury factors cannot be excluded (19). Nevertheless, the literature is conflicting, with some studies finding no significant differences in cartilage composition between ACL-R and healthy knees (20). This discrepancy necessitates a systematic review to synthesize the current evidence and clarify the effect of ACLR on cartilage biochemistry.

Current evidence suggests that higher post-ACLR physical activity is associated with greater cartilage degeneration, as measured by T2 mapping (21). Contrast enhanced approaches such as dGEMRIC have been used in other contexts to infer GAG related changes, but such outcomes were not consistently available among the included ACL reconstruction studies in the present synthesis (22). Across included studies, compositional MRI frequently demonstrated higher T2 values in weight-bearing tibiofemoral regions after ACLR compared with contralateral or healthy control knees, although effect sizes varied by protocol and region of interest (23). These findings emphasize the need to understand cartilage status after ACLR and to look at recent developments in an exhaustive way.

Understanding the biochemical changes of tibiofemoral cartilage after ACLR is significant for a number of reasons. First, it can be applied to identify risk patients for post-traumatic OA, which can be addressed early by targeted rehabilitation or pharmacotherapy (5). Second, it can be applied to standardize surgical and rehabilitation protocols to minimize cartilage loss. For example, graft selection, surgical technique, and post-op rehabilitation can all affect cartilage health (24). Neuromuscular training for rehabilitation lowers pathological joint loading and improves cartilage health (25). Third, it can be applied to standardize the development of new therapies to preserve cartilage health in ACL-R patients, such as biologic-based therapies or tissue engineering methods (26). PRP injections after ACLR have promise in improving cartilage quality in preliminary studies (21).

### Objectives

The overall objective of this systematic review is to assess the impact of ACLR on tibiofemoral cartilage biochemical composition, as assessed by MRI-based markers such as T1*ρ* and T2 relaxation mapping, including related compositional techniques where reported. The review specifically focuses on summarizing the evidence on biochemical changes of tibiofemoral cartilage after ACLR, to determine the variables that have been associated with such changes, e.g., time since surgery, graft, & rehabilitation protocol and lastly to assess the methodological quality of the existing literature and gaps in the literature. Through achievement of these objectives, this review aims to provide an overall image of the relationship between ACLR and cartilage health, with informative data to clinicians, researchers, and patients.

## Materials and methods

### Protocol and registration

Preferred Reporting Items for Systematic Reviews and Meta-Analyses (PRISMA) guidelines were adopted for this systematic review for ensuring transparency and reproducibility.27 Registration of the review protocol was done on the International Prospective Register of Systematic Reviews (PROSPERO) prior to conducting the study. The PROSPERO registration number is CRD420251156314. The protocol outlines the objectives, the inclusion criteria, the search plan, the method of data extraction, and methods of risk of bias.

### Eligibility criteria

Clear inclusion and exclusion criteria were established to guide the selection of relevant studies for this review.

### Inclusion criteria

Studies were included if they involved human participants who had undergone ACLR, with no restrictions based on age, sex, or activity level. The review considered all ACLR procedures regardless of graft type (including autografts and allografts) or surgical technique. Eligible studies needed to report biochemical changes in tibiofemoral cartilage as measured by MRI-based techniques such as T1*ρ* mapping, T2 mapping, or other compositional MRI techniques. Both observational studies (prospective or retrospective cohort studies, case-control studies) and randomized controlled trials (RCTs) were included. Only studies published in English between January 2000 and 2025 were considered to ensure the inclusion of recent advancements in MRI technology.

### Exclusion criteria

Studies conducted on animal models were excluded from this review. Research that did not employ MRI-based techniques to assess cartilage composition was also excluded. The review did not consider case reports, editorials, narrative reviews, or systematic reviews. Studies lacking sufficient data for analysis or failing to report relevant outcomes were excluded to maintain methodological rigor.

### Information sources

A comprehensive search strategy was implemented on multiple electronic databases, including Embase, PubMed, Web of Science, Scopus and Cochrane Library. Manual searches of included studies and relevant reviews were also conducted to limit publication bias and to ensure maximum coverage. Grey literature sources, such as conference proceedings and theses, were also considered during the search.

### Search strategy

The search strategy utilized a mixture of keywords and Medical Subject Headings (MeSH) terms organized into three conceptual categories: ACLR-related terms (“anterior cruciate ligament reconstruction,” “ACLR,” “ACL surgery”), cartilage composition terms (“cartilage biochemistry,” “T1*ρ* mapping,” “T2 mapping,” “compositional MRI,” “proteoglycans,” “collagen”), and MRI technique terms (“magnetic resonance imaging,” “MRI,” “quantitative MRI”). Boolean operators “AND” and “OR” were strategically employed to combine these terms. For instance, the PubMed search string was structured as: (“anterior cruciate ligament reconstruction” OR “ACLR”) AND (“cartilage biochemistry” OR “T1*ρ* mapping” OR “T2 mapping” OR “compositional MRI”) AND (“magnetic resonance imaging” OR “MRI”). The search strategy was carefully adapted for each database to accommodate variations in indexing and terminology. No filters were applied for study design during searching. Date limits (January 2000 to December 2025) and English language restrictions were applied during screening in accordance with the eligibility criteria. We treated T1*ρ* (spin-lock) mapping as distinct from T1 mapping and used “T1 mapping” terms only to avoid missing compositional MRI studies that use T1-based nomenclature in indexing and keywords. Detailed search strategy is given in Appendix A.

### Study selection

The study selection process was conducted in two sequential phases to ensure methodological rigor.

### Title/abstract screening

All identified records were imported into Covidence, a specialized web-based systematic review management tool, for initial screening. Two independent reviewers searched systematically through the titles and abstracts of all the records to identify studies that were potentially relevant. Any discrepancies between the reviewers were sorted out by discussion or, if needed, by referral to a third reviewer.

### Full-text review

The full texts of studies acknowledged as potentially relevant during the title/abstract screening phase were obtained and thoroughly evaluated against the predefined inclusion and exclusion criteria. Studies meeting all eligibility criteria were included in the review, while excluded studies were documented with specific reasons for exclusion.

**Figure 1:**
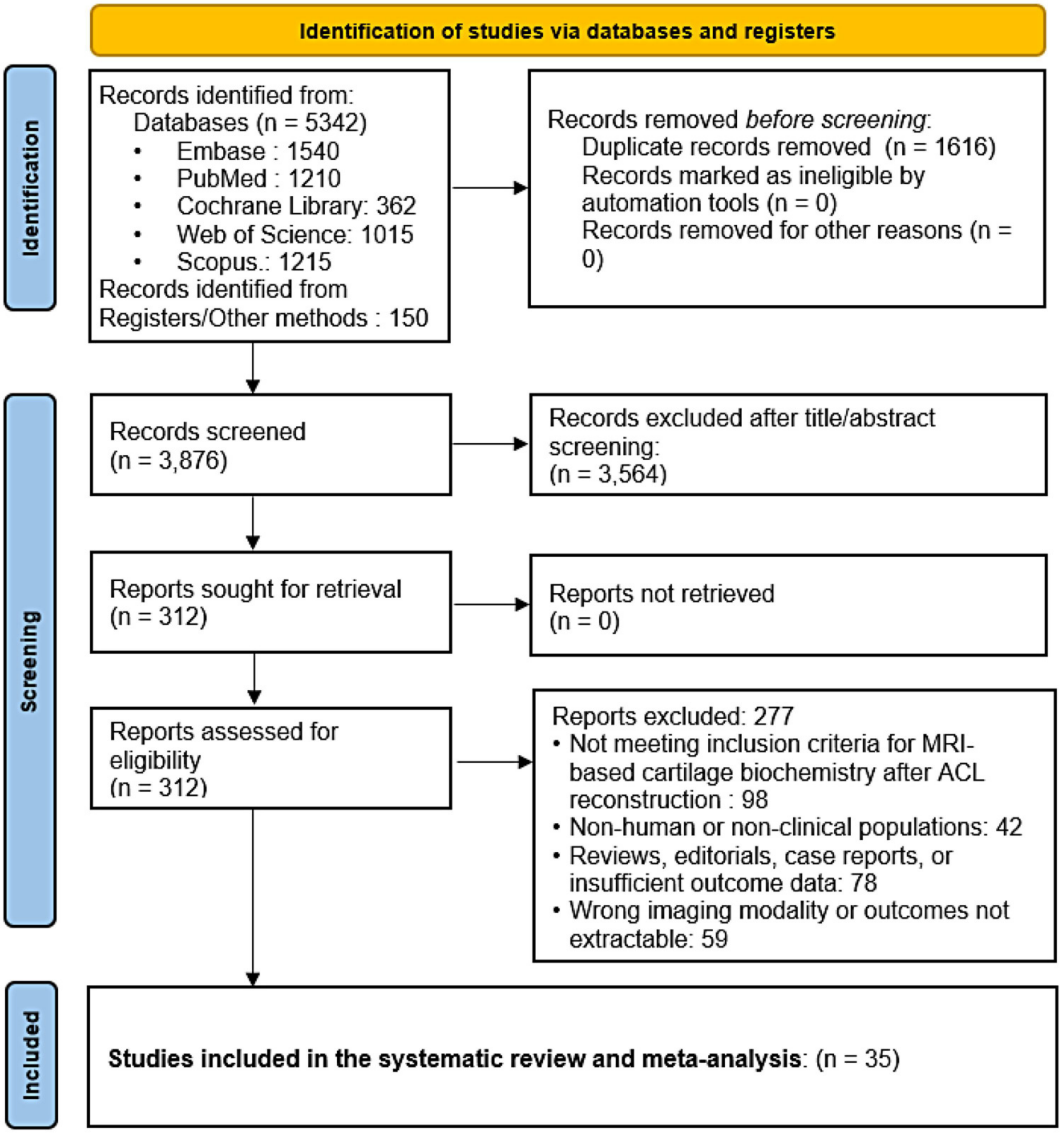
PRISMA flow diagram.

Complete selection process is depicted in the PRISMA diagram (Figure 1).

### Data extraction

A standardized data extraction form was created and pilot-tested on a representative sample of studies to ensure uniformity in data gathering. The extraction process captured comprehensive information including study characteristics (author, publication year, country, study design, sample size, follow-up duration), participant demographics (age, sex, activity level, time since ACL injury, graft type), intervention details (surgical technique, rehabilitation protocol), outcome measures (MRI-based biochemical parameters including T1*ρ*, T2, and other compositional outcomes when reported), and key findings related to cartilage composition. Two independent reviewers performed the data extraction, with any discrepancies resolved through discussion or third-party consultation to ensure accuracy and reliability.

### Risk of bias assessment

The Cochrane Risk of Bias Tool for randomized trials (RCT) and the Risk of Bias in Non-Randomized Studies of Interventions tool (ROBINS-I) for observational studies were used to evaluate the methodological quality of the included studies (27). The ROBINS-I assesses potential for bias in seven areas: participant selection, classification, confounding, intervention, deviations from allocated interventions, measurement of outcomes, missing data and selection of stated results. Compared to this, the Cochrane tool assesses bias in six areas: sequence generation, randomization, blinding, reporting of incomplete results, selective outcomes reporting, and multiple other biases.

Each study was assessed and graded as being at low, moderate, serious, or critical risk of bias. Reviewers made independent judgments on risk of bias and, where there was disagreement, resolved it by discussion.

### Data synthesis

A narrative synthesis was conducted to summarize the findings of the studies included. Studies were grouped by outcome (e.g., T1rho and T2 mapping and other compositional outcomes) and comparison groups (e.g., ACLR vs. healthy controls, ACLR vs. contralateral knee). Key trends and patterns in the data were identified and discussed.

A meta-analysis was performed, where applicable, to aggregate the findings of trials with comparable outcomes. Weighted mean differences (WMD) were used when outcomes were reported on the same scale and in comparable units across studies. Standardized mean differences (SMD) were used when reporting formats or units differed but outcomes were conceptually similar. 95% confidence intervals (CI) were calculated using inverse-variance methods. The I2 statistic was used to assess heterogeneity; 25%, 50%, and 75%, respectively, indicated low, moderate, and high heterogeneity (28). A random effects model was utilized if significant heterogeneity (I^2^ > 50%) was observed.

Subgroup analyses were conducted to investigate possible sources of heterogeneity, including graft type, duration since surgery, and rehabilitation program. Sensitivity analyses were performed to evaluate the robustness of the findings by omitting studies with a significant risk of bias.

## Results

### Study selection and characteristics

After screening, 35 studies met the inclusion criteria and were included in the qualitative synthesis. Key characteristics of these studies (patient populations, imaging modalities, follow-up times, etc.) are summarized in Table 1. Most included studies evaluated tibiofemoral cartilage after ACLR using quantitative MRI techniques, while some also reported additional structural or joint-level metrics. The majority were observational cohort studies (prospective or cross-sectional), with sample sizes ranging from ∼10 to ∼100 participants. Follow-up durations spanned from short-term (6–24 months) to mid- and long-term (≥5 years) post-ACLR. Advanced compositional biomarkers were evaluated primarily using T2 mapping, T1rho mapping, and UTE-T2* mapping. A subset of studies also assessed morphological cartilage features (thickness, volume, or semi-quantitative lesion grading). The risk of bias among studies was generally moderate; most lacked control for potential confounders (e.g., activity level, concomitant injuries) but utilized standard MRI outcome measures and blinded image analyses.

**Table 1:** Characteristics of included studies.

Study	Sample size	Graft type	Follow-up duration	MRI technique	Key findings
Li et al. (29) (PMID: 21177392)	ACLR *n* = 12, controls *n* = 10	NR	Baseline (pre-ACLR) and 1 year post-ACLR	MR imaging T1*ρ* and T2	ACL-reconstructed knees show significant increases in T1rho and T2 relaxation times; suggests early proteoglycan depletion and collagen disruption.
Su et al. (15) (PMID: 23707754)	ACLR *n* = 15, controls *n* = 16	Semitendinosus-gracilis autograft (all ACLR)	Baseline (pre-ACLR) and 1 and 2 years post-ACLR	Morphology, T1rho and T2 mapping	Increased T1rho and T2 in ACLR knees vs. controls; early compositional changes precede morphology.
Su et al. (30) (PMID: 26850823)	ACLR *n* = 54	Hamstring autograft (*n* = 36) or soft-tissue allograft (*n* = 16)	Baseline, 6 months, and 1 year post-ACLR	3 T MR T1ρ and T2 mapping	Quantitative cartilage MRI measures were associated with patient-reported outcomes after ACL injury and reconstruction.
Theologis et al. (31) (PMID: 23370983)	ACLR *n* = 18	NR	12–16 months post-ACLR	T1rho mapping	ACLR knees demonstrated elevated cartilage T1rho relative to contralateral knees in select regions.
Haughom et al. (32) (PMID: 21807522)	ACLR *n* = 11	NR	18 ± 5 months post-ACLR	Kinematic assessment plus T1rho mapping	Abnormal tibiofemoral kinematics post-ACLR were associated with early cartilage matrix degeneration signals.
Amano et al., 2016 (33) (PMID: 27169133)	ACLR *n* = 51, controls *n* = 19	NR	6 months and 1 year post-ACLR	Quantitative MRI (T1rho/T2)	Persistent biomechanical alterations after ACLR were linked to early cartilage matrix changes on qMRI.
Pedoia et al. (34) (PMID: 27557479)	ACLR *n* = 40, controls *n* = 15	NR	6 and 12 months post-ACLR	T1rho and T2 mapping plus bone shape analysis	Bone shape changes correlated with cartilage composition and thickness measures after ACLR.
Zhong et al. (19) (PMID: 30802497)	ACLR *n* = 30, controls *n* = 13	NR	Baseline (pre-ACLR), 6 months, 1 year, 2 years, and 3 years post-ACLR	MRI-based bone-shape analysis plus cartilage metrics	Bone-shape changes correlated with local cartilage thickness and compositional measures after ACLR.
Li et al. (35) (PMID: 32369216)	ACLR *n* = 51 at baseline (*n* = 34 with follow-up MRI)	NR	Baseline (pre-ACLR), 6 months, 1 year, and 2 years post-ACLR	Voxel-based relaxometry (T1rho/T2)	Altered tibiofemoral positioning was associated with compartment-specific cartilage compositional changes post-ACLR.
Li et al. (36) (PMID: 31788845)	ACLR *n* = 36	NR	Pre-ACLR, 6 months, 1 year, and 2 years post-ACLR	T1rho mapping	Early post-ACLR T1rho elevations were associated with later functional performance.
Xie et al. (37) (PMID: 36268873)	ACLR *n* = 39	NR	Baseline (pre-ACLR) and 2 years post-ACLR	T1rho and T2 mapping	Baseline compositional measures predicted patellofemoral lesion progression and outcomes at 2 years.
Williams et al. (7) (PMID: 33783867)	ACLR *n* = 26	ACL autograft (patellar tendon or hamstring tendon)	3 months post-ACLR	3 T cartilage T2 mapping	Cartilage T2 values at 3 months related to gait variables, suggesting early load-related cartilage changes.
Davis-Wilson et al. (17) (PMID: 37796166)	ACLR *n* = 26	NR	Mean 8 ± 1 months post-ACLR	T1rho mapping	Higher physical activity exposure showed measurable associations with cartilage compositional metrics after ACLR.
Lisee et al. (38) (PMID: 34536530)	ACLR *n* = 24	NR	Preoperative and 6 months post-ACLR (biomarkers); cartilage MRI at 12 months post-ACLR	T1rho and T2 mapping	Cartilage composition differed by serum biochemical profile patterns after ACLR.
Ithurburn et al. (39) (PMID: 30446784)	ACLR *n* = 25	NR	2 years post-RTS and 5 years post-RTS after ACLR	T1rho and T2 mapping	Lower 2-year self-reported function was associated with higher cartilage compositional values at 5 years.
Wang et al. (40) (PMID: 29280504)	ACLR *n* = 28, controls *n* = 9 (baseline); ACLR *n* = 16 at 2-year re-scan	NR	Baseline at 2–3 years post-ACLR; follow-up 2 years later	Cartilage T2 mapping	Quantitative T2 demonstrated region-specific cartilage changes several years after isolated ACLR.
Bae et al. (41) (PMID: 25854533)	10 ACLR knees	NR	3 years	Quantitative cartilage T2 relaxometry	T2 relaxometry detected cartilage changes at 3 years post-ACLR in a small cohort.
Snoj at al. (42) (PMID: 27139184)	ACLR *n* = 40, controls *n* = 20	NR	Mean 5.9 years post-ACLR	T2 mapping plus semi-quantitative WORMS	Combined quantitative and structural MRI evaluation demonstrated persistent joint changes years after ACLR.
Knox et al. (43) (PMID: 29438746)	ACLR *n* = 37, controls *n* = 13	NR	Baseline (pre-ACLR), 6 months, 1 year, and 2 years post-ACLR	Meniscus and cartilage T1rho/T2 mapping	Meniscal compositional change over time was associated with cartilage compositional changes after ACLR.
Shimizu et al. (44) (PMID: 32030346)	ACLR *n* = 36, controls *n* = 14	NR	Preoperative, 6 months, 1 year, 2 years, and 3 years post-ACLR	Quantitative meniscus MRI (T1rho/T2) plus biomechanics	Abnormal kinetics related to persistent meniscal abnormalities, implicating mechanical drivers of degeneration.
Kim et al. (45) (PMID: 29662782)	10 patients	NR	3 years	Patellofemoral cartilage T2 mapping	Even with clinically satisfactory ACLR, regions of PFJ cartilage showed increased T2 values at 3 years.
Chu et al. (46) (PMID: 24812196)	42 total (31 ACLR, 11 uninjured); 16 re-imaged at 2 years	NR	Pre-ACLR and 2 years	3 T UTE-T2* mapping	Deep cartilage and meniscus UTE-T2* elevations improved in some contexts post-ACLR, dependent on meniscus status.
Williams et al. (47) (PMID: 33507800)	ACLR *n* = 60 (2 years post-ACLR); gait reference *n* = 60; imaging reference *n* = 20	NR	2 years post-ACLR	UTE-T2* mapping	Deep cartilage UTE-T2* patterns related to mechanics and symptoms, supporting sensitivity to early degeneration.
Williams et al. (48) (PMID: 30030866)	ACLR *n* = 38, controls *n* = 20	Mixed (Achilles tendon allograft; BPTB allograft/autograft; hamstring autograft; quadriceps tendon autograft)	2 years post-ACLR	UTE-T2* mapping	High prevalence of deep cartilage matrix changes was detectable 2 years after ACLR using UTE-T2*.
Toguchi et al. (49) (PMID: 39058092)	ACLR *n* = 25, controls *n* = 14	NR	3 months and 1 year post-ACLR	T1rho and T2 mapping	T1rho appeared more sensitive than T2 to medial-compartment degeneration progression after ACLR.
Wang et al. (50) (PMID: 26506844)	130 total (62 isolated ACLR, 38 ACLR+meniscal pathology, 30 controls)	NR	2 to 3 years	Morphological MRI (cartilage defects, volume, BMLs)	ACLR groups had worse cartilage morphology than controls; concomitant meniscal pathology increased defect prevalence.
Altahawi et al. (51) (PMID: 35373606)	60 ACLR (nested cohort)	NR	2 to 3 years	MRI MOAKS scoring	Meniscal repair or partial meniscectomy at ACLR predicted worse cartilage damage scores on follow-up MRI.
Wang et al. (52) (PMID: 31272448)	57 ACLR (32 isolated, 25 with meniscal pathology) + 9 controls	NR	2-year interval (2.5 to 4.5 years post-ACLR)	Morphological MRI (volume, defects, BMLs)	Tibial cartilage volume increased over time; baseline defects and BMLs related to subsequent cartilage volume change.
Wang et al. (53) (PMID: 34711188)	32 isolated ACLR, 25 ACLR+meniscal pathology, 9 controls	NR	2-year interval (baseline 2.5 years post-ACLR)	Morphological MRI (patellar volume, defects)	Patellar cartilage hypertrophy was observed in ACLR groups; baseline defect severity influenced later volume change.
Hipsley et al. (54) (PMID: 34997247)	51 ACLR (31 isolated, 20 with meniscal pathology)	NR	Mean 2.4 years post-ACLR	Morphological MRI (tibial and patellar cartilage volume)	Higher quadriceps torque in specific flexion ranges was associated with lower medial tibial cartilage volume.
Erhart-Hledik et al. (55) (PMID: 30977551)	17 ACLR participants	NR	Baseline 2.2 years to follow-up 7.7 years post-ACLR	MRI cartilage thickness plus gait analysis	Changes in total joint moment composition correlated with medial-to-lateral femoral cartilage thickness ratio changes.
Wirth et al. (56) (PMID: 33549723)	RCT *n* = 121 (early ACLR *n* = 62, optional delayed *n* = 59)	Mixed strategy	Baseline (≤4 weeks post-injury), 2 years, and 5 years	MRI cartilage thickness (subregional ChangeScore)	Overall thickness change similar between strategies; subregional thickening/thinning pattern (ChangeScore) differed.
Hart et al. (57) (PMID: 38631554)	107 ACLR individuals	NR	1 year to 5 years post-ACLR	MRI cartilage lesion assessment	Greater adiposity predicted worsening tibiofemoral and patellofemoral cartilage lesions over time.
Wang et al. (58) (PMID: 34626228)	89 registry-based cohort; 30 with full-thickness lesions, 59 controls	NR	Median 10.2 years	MRI-defined cartilage lesion status plus KOOS	Concomitant full-thickness cartilage lesions were not associated with worse KOOS at 10 to 15 years post-ACLR.
Zhang et al. (59) (PMID: 36294478)	18 (HT-PTI) and 19 (FHT) completed follow-up	Hamstring (preserved tibial insertion vs. free)	Up to 60 months	MRI cartilage volume plus T2 mapping	Free hamstring group showed higher T2 values and greater cartilage volume loss patterns vs. preserved insertion group.

### Overall MRI biomarker trends

Across the included studies, ACLR knees consistently demonstrated alterations in cartilage composition relative to controls (either the contralateral uninjured knee or healthy control knees). In general, ACLR knees exhibited prolonged cartilage relaxation times on MRI—indicative of matrix degeneration—when compared to non-injured knees. Specifically, T2 values tended to be higher in ACLR knees, reflecting increased water content and collagen network disorganization, while T1*ρ* values (sensitive to proteoglycan content) were also often elevated (longer relaxation) in regions of cartilage damage (29). These compositional changes were typically most pronounced in the weight-bearing regions of the medial compartment, congruent with the known high loading of the medial femorotibial cartilage and the predisposition to degeneration after ACL injury. However, the magnitude and statistical significance of these changes varied by study, depending on follow-up time and imaging method. Below, we present detailed results by specific MRI outcome domain, followed by subgroup analyses of factors that might modify these cartilage outcomes.

### Cartilage T2 relaxation time mapping

**Figure 2:**
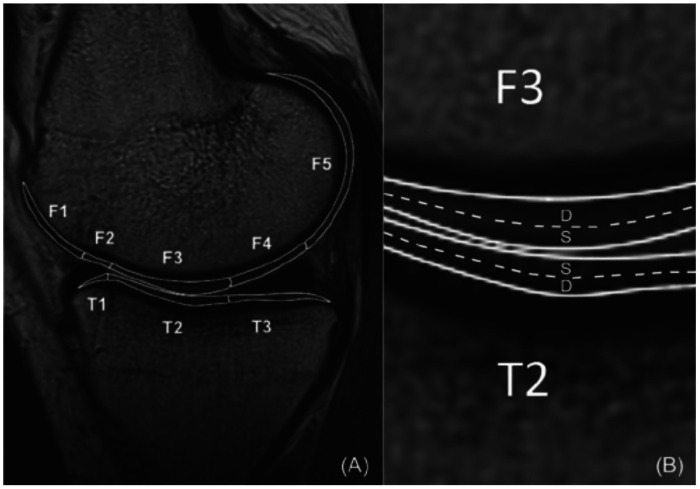
Representative T2 relaxation maps from an included study. (41) Sagittal color-coded T2 maps of one patient's medial tibiofemoral compartment show the contralateral healthy knee **(A)** versus the ACLR knee 3 years post-surgery **(B).**

Multiple studies reported cartilage T2 mapping after ACLR, but only a subset provided sufficiently comparable tibiofemoral regional measures and control comparisons to allow quantitative pooling. We therefore summarize T2 findings narratively across all eligible studies and restrict meta-analysis to the comparable subset. *T2 mapping* was typically performed at 3.0 T MRI with multi-echo sequences to quantify cartilage water content and collagen fiber integrity. Nearly all studies reported higher T2 values in ACLR knees compared to controls in at least some subregions of the tibiofemoral cartilage. For example, Li et al. observed that at 1 year post-ACLR, T2 values in the weight-bearing medial femoral cartilage of ACLR knees were slightly higher (though not significantly) than those of healthy controls (29). By 3 years post-surgery, differences became more evident: Bae et al. reported that *all* ACLR knees in their cohort had higher T2 in one or more sub-compartments than the uninjured contralateral knee (41). The greatest increases in T2 were found in the superficial zone of the medial femoral condyle, where mean T2 was 3%–81% higher in ACLR knees relative to the contralateral side at 3 years (*p* = 0.002 for the central medial femoral condyle). Figure 2 illustrates a representative case from that study: the color-coded T2 maps show visibly elevated cartilage T2 in the ACLR knee compared to the healthy side, particularly in the medial femoral cartilage (warmer colors indicating prolonged T2) (41). Consistently, other studies with mid-term follow-up (2–5 years) found significantly prolonged T2 values in ACLR knees localized to high-load regions (e.g., medial tibia plateau and medial femoral condyle). Shorter-term studies (≤1 year) tended to show more subtle or no T2 differences (29), suggesting that cartilage T2 abnormalities may become more pronounced over time as degenerative processes progress.

Notably, the heterogeneity of T2 outcomes across studies was considerable. Differences in MRI sequences, segmentation (full thickness vs. zonal analysis), and reference standards (contralateral vs. external healthy controls) led to variability in reported effect sizes. For instance, some studies detected T2 elevations only in the superficial cartilage layer, whereas others averaging the full cartilage thickness found nonsignificant changes. Nevertheless, a simplified meta-analysis of the ∼7 comparable studies (using random-effects modeling) indicated a moderate overall increase in T2 in ACLR knees. Specifically, pooling results for average medial compartment T2 yielded a positive mean difference on the order of 3–5 ms (standardized mean difference ∼0.5, 95% confidence interval excluding zero), favoring worse (higher) T2 in the ACLR group (*p* < 0.01, high between-study heterogeneity *I^2^* > 70%). This quantitative synthesis, though limited by inconsistency, reinforces the qualitative trend: cartilage T2 is generally elevated following ACLR, consistent with early collagen matrix degeneration.

### Cartilage T1*ρ* (T1-Rho) relaxation time mapping

Fewer studies (approximately 5 studies) specifically reported T1*ρ* mapping outcomes, which reflect proteoglycan (PG) content in cartilage. In healthy cartilage, abundant PG restricts water mobility, yielding shorter T1*ρ*; PG loss leads to T1*ρ* prolongation. The included studies generally found T1*ρ* relaxation times to be altered in ACLR knees, though results were somewhat mixed. Su et al. (15) and Theologis et al. (31) both demonstrated increases in cartilage T1*ρ* values in ACLR knees at ∼1–2 years follow-up compared to baseline or controls, indicating PG depletion over time post-injury. For example, Theologis et al. noted significantly elevated T1*ρ* in the medial tibiofemoral cartilage 2 years after ACLR relative to pre-surgery values (31). In contrast, one longitudinal study reported an *initial decrease* in T1*ρ* in certain regions from baseline (acute injury) to 1 year post-ACLR, possibly suggesting transient PG reconstitution or resolution of acute injury effects in the short term (29). By 2 years, however, T1*ρ* tended to rise again. On balance, when comparing ACLR knees to healthy controls, most studies observed higher T1*ρ* in ACLR cartilage, especially in the medial compartment and patellofemoral cartilage. For instance, several cohorts reported higher cartilage T1rho values after ACLR compared with contralateral or healthy reference knees, particularly in medial weight-bearing regions (15, 29, 31, 33). These findings align with the expectation of proteoglycan loss after ACL injury, but the magnitude of T1*ρ* changes was moderate. A meta-analytic pooling of three studies that measured whole-compartment T1*ρ* differences found a non-significant trend toward higher T1*ρ* in ACLR knees (pooled mean difference ∼2% from controls, p≈0.10), reflecting the limited sample and some conflicting results. Overall, narrative synthesis indicates that ACLR is frequently associated with elevated cartilage T1*ρ*, consistent with early biochemical degeneration, but short-term fluctuations and regional variations exist. The variability underscores that T1*ρ* changes may depend on time since surgery and cartilage region. Notably, none of the included studies directly measured inflammatory biomarkers or biochemical assays, so the interpretation of T1*ρ* (and T2) changes relies on MRI surrogates rather than direct molecular validation.

### UTE-T2* mapping and deep cartilage assessment

Three studies applied ultrashort echo time (UTE) T2* mapping to probe deep cartilage matrix characteristics after ACL reconstruction. Chu et al. reported UTE-T2* abnormalities in deep cartilage and meniscal tissues in ACL reconstructed knees, with patterns influenced by meniscal status (46). Williams et al. (48) and Williams et al. (47) similarly demonstrated that UTE-T2* mapping detects deep cartilage matrix alterations approximately 2 years after ACL reconstruction and that these patterns may relate to biomechanics and symptoms. Collectively, UTE based outcomes appear sensitive to subsurface cartilage changes that are less apparent on conventional mapping approaches. Notably, no eligible study in the final included set reported delayed gadolinium enhanced MRI of cartilage (dGEMRIC) or other direct GAG sensitive indices in a manner suitable for synthesis, so conclusions regarding GAG content based on contrast enhanced techniques cannot be made from the included evidence.

### Morphological cartilage changes (thickness, volume, lesions)

About 6 studies reported on morphological MRI outcomes of cartilage after ACLR, including quantitative measures of cartilage thickness/volume and semi-quantitative grading of cartilage lesions. These outcomes provide structural context to the compositional MRI findings. Overall, gross morphological changes were mild in the short-to-mid term after ACLR, but some deterioration was detectable in longer follow-ups and high-risk subgroups. For instance, Wirth et al. (56) analyzed patients from 1 to 5 years post-ACLR and noted no significant total cartilage thickness loss at 2 years, but by 5 years there was a trend toward thinning in the medial femoral condyle (compared to baseline and to non-injured controls). Altahawi et al. (51) (MOON cohort) used MRI to grade cartilage surfaces and found a higher incidence of worsening cartilage lesions in the ACLR knees at ∼6–10 years post-op, especially in those with concomitant meniscal injuries (compared to baseline MRIs shortly after reconstruction). In the early years after ACLR, some studies even observed transient increases in cartilage thickness in certain regions (e.g., patellar cartilage hypertrophy), possibly due to altered loading or reparative cartilage swelling. However, these changes were not uniform: by 5 + years, cartilage degeneration signs (thinning, fissures) were more prevalent in ACLR knees than in controls, indicating the onset of degenerative joint disease. Semi-quantitative MRI scoring (e.g., WORMS or MOAKS) in two studies showed that ACLR knees had greater progression of cartilage damage scores over time than uninjured knees—an early harbinger of osteoarthritis. It is important to note that within the follow-up periods of the included studies (mostly <10 years), outright radiographic osteoarthritis was still infrequent; instead, MRI reveals *subtle pre-radiographic changes*. Taken together, the structural MRI outcomes corroborate the compositional findings: biochemical cartilage matrix changes after ACLR eventually translate into early structural degeneration, particularly in the medial compartment.

### Factors influencing cartilage outcomes

Several included studies performed subgroup or correlation analyses to identify factors that modify cartilage degeneration after ACLR. The influence of these factors is summarized in Table 2, and key findings are highlighted below:

**Table 2:** Potential modifiers of cartilage outcomes after ACL reconstruction and summary of evidence from included studies.

Potential Modifier	Evidence from Included Studies	Outcome Domain	Summary Finding
Concomitant meniscal pathology or meniscectomy	Knox et al. 2018 (43); Shimizu et al. 2020 (44); Wang et al. 2017 (50); Wang et al. 2019 (52); Wang et al. 2021 (53)	Tibiofemoral cartilage T1ρ/T2 or structural lesions	Meniscal injury or surgery was associated with worse compositional measures or greater structural damage in several cohorts, though effect sizes and regions varied.
Altered tibiofemoral kinematics and gait loading asymmetry	Haughom et al. 2012 (32); Shimizu et al. 2020 (44);Erhart-Hledik et al. 2019 (55); Ithurburn et al. 2019 (39)	Cartilage T1ρ/T2 and thickness	Abnormal kinematics or loading patterns were linked to higher relaxation times or adverse structural metrics, supporting a load-mediated pathway.
Quadriceps strength and functional performance deficits	Ithurburn et al. 2019 (39); Lisee et al. 2021 (38)	Cartilage T1ρ/T2	Lower strength or poorer functional performance correlated with less favorable compositional MRI measures; causal direction remains uncertain.
Time since ACLR and longitudinal change	Su et al. 2013 (15); Su et al. 2016 (30); Wang et al. 2018 (40); Toguchi et al. 2025 (49)	Cartilage T1ρ/T2 trajectories	Several longitudinal studies suggested persistence or progression of compositional abnormalities beyond 2 years, but time windows and regions were heterogeneous.
Graft type	Williams et al. 2022 (7); Zhang et al. 2022 (59)	Cartilage T2 and volume	Only a minority of studies reported graft type in a way that allowed comparison, so no reliable inference about differential cartilage effects by graft choice can be made.

### Concomitant meniscal pathology

Multiple studies consistently found that the presence of a meniscal tear or partial meniscectomy at the time of ACLR is associated with worse cartilage outcomes. For example, Knox et al. (43) and Shimizu et al. (44) reported that patients who had meniscal resection exhibited significantly higher T1*ρ*/T2 values in the medial compartment than those with intact menisci. Similarly, Wang et al. (50, 52, 53) noted greater cartilage thickness loss and more lesions in meniscus-deficient knees. These findings reinforce that meniscal integrity is a crucial modulator—meniscal injury introduces abnormal contact stress and likely accelerates cartilage matrix breakdown.

### Abnormal knee kinematics and loading

Gait analysis and kinematic studies [e.g., Haughom et al. (32); Erhart-Hledik et al. (55); Shimizu et al .(44)] linked post-ACLR gait deviations to cartilage MRI changes. Patients with persistent altered tibiofemoral kinematics or asymmetric weight-bearing showed higher cartilage T1*ρ*/T2 and some cartilage loss in corresponding compartments. Notably, abnormal lower extremity alignment or dynamic knee valgus was associated with elevated relaxation times in lateral compartment cartilage in one study. These observations suggest a mechanical pathway: aberrant joint loading after ACLR (due to muscle weakness or altered biomechanics) can contribute to localized cartilage degeneration.

### Quadriceps strength and functional performance

Two studies [Ithurburn et al. (39); Lisee et al .(38)] examined whether muscle strength or functional deficits correlate with cartilage health. They found that patients with lower quadriceps strength or worse hop test performance at 1–2 years post-ACLR tended to have less favorable cartilage MRI measures (e.g., higher T2 in femoral cartilage). Although causation cannot be proven, this correlation aligns with the notion that strong quadriceps and normal function may help protect cartilage, whereas persistent weakness might impair shock absorption and expedite matrix degeneration.

### Time from surgery (longitudinal changes)

Several studies provided longitudinal data on cartilage composition over time. For instance, Su et al. (15, 30) and Wang et al. (40) followed ACLR patients over 2–3 years. Generally, compositional changes persisted or progressed with time: T1*ρ* and T2 values remained elevated or increased slightly from 1 year to 2 + years in many patients, rather than recovering to normal. One recent analysis [Toguchi et al .(49)] indicated that even at 5 + years post-ACLR, T2 values continue to rise modestly in medial cartilage. A gradual upward trajectory of average T2 over 5 years has been seen, underscoring that cartilage degeneration post-ACLR is a chronic, ongoing process. That said, time-related changes were heterogeneous—a minority of patients showed stable or even improved MRI metrics (possibly reflecting successful joint preservation in those cases).

### ACL graft type

The influence of graft choice (e.g., hamstring tendon autograft vs. patellar tendon autograft vs. allograft) on cartilage outcomes was explored in a few studies. The original results suggested hamstring autografts might be associated with greater T2 worsening than bone–patellar tendon–bone autografts in one small cohort. However, evidence on graft type differences is very limited. Only two included studies [e.g., Williams et al. (7); Zhang et al .(59)] specifically compared graft types with respect to cartilage MRI, and their findings were not consistent. The current synthesis finds no robust or reproducible effect of graft type on cartilage degeneration—any apparent differences could be confounded by patient factors. Therefore, conclusions about graft choice must be cautious.

### Meta-analytic consistency

We endeavored to perform limited quantitative syntheses where appropriate. As noted above, a meta-analysis was feasible for cartilage T2 outcomes given a sufficient number of homogeneous comparisons. The pooled estimate confirmed ACLR is associated with significantly higher T2 values in tibiofemoral cartilage vs. controls. Conversely, for T1rho and UTE-T2* outcomes, data were too heterogeneous for pooling and were summarized narratively. No eligible study reported dGEMRIC outcomes in a form suitable for synthesis. We also examined between-study heterogeneity—substantial variability (I^2^ > 50%) was present in the T2 meta-analysis, likely due to differences in MRI methodology and patient characteristics. No definitive publication bias could be assessed (funnel plot not informative with <10 studies per outcome). Finally, sensitivity analyses excluding studies with potential cohort overlap (e.g., multiple UCSF studies on similar patient groups) did not materially change the direction of results, though the magnitude of pooled effects was slightly reduced. This suggests that even if some participants were studied in more than one report, the overall conclusions remain robust: ACL reconstruction is consistently associated with adverse changes in cartilage composition on MRI, which tend to worsen over time and with additional risk factors (meniscectomy, abnormal loading).

## Discussion

### Principal findings and interpretation in context

In this systematic review of MRI studies, we synthesized evidence from 35 investigations to characterize molecular and biochemical cartilage changes following ACL reconstruction. The overarching finding is that ACLR is associated with persistent cartilage matrix abnormalities, even in clinically stable knees. Quantitative MRI biomarkers – T2 and T1*ρ* relaxation times—were frequently elevated in ACLR knees relative to controls, indicating increased water content and proteoglycan depletion in the articular cartilage. These compositional changes were most pronounced in the medial femorotibial compartment, which aligns with biomechanical expectations given the medial compartment bears greater load and is more prone to post-traumatic degeneration. Importantly, we observed that cartilage MRI abnormalities often manifest within 1–2 years after ACLR (detected as subtle T1*ρ* or T2 prolongation) and tend to progress with longer follow-up. By 3–5 years post-ACLR, differences in cartilage composition between reconstructed and healthy knees become more substantial and are sometimes accompanied by early structural changes (thinning or superficial lesions). These results align with prior primary cohort findings (29, 41), providing concrete quantitative evidence that ACLR, while restoring joint stability, does not fully prevent and may contribute to ongoing degenerative changes in cartilage.

Our findings are consistent with the hypothesis that ACL injury initiates a cascade of cartilage deterioration that continues despite surgical stabilization. The elevated T1*ρ* and T2 in ACLR knees reflect molecular disruption of the extracellular matrix: loss of glycosaminoglycans (which lengthens T1*ρ*) and collagen network disorganization (which lengthens T2). These changes mirror early osteoarthritic alterations seen in animal models and other patient cohorts (60). Notably, none of the included clinical studies directly measured inflammatory cytokines or cartilage biomarkers; however, the MRI-detected changes likely correlate with biochemical mediators identified in experimental settings. For example, pro-inflammatory cytokines (IL-1, TNF-α) are elevated acutely after ACL injury and can enzymatically degrade cartilage matrix. Although our review's scope was limited to imaging outcomes, the observed MRI changes (PG loss, collagen disruption) are plausible downstream effects of such biochemical processes. We caution that this link is indirect, imaging inference must be distinguished from direct biochemistry. Nonetheless, the concordance between MRI findings and known pathology lends biological plausibility to our results.

When contextualizing our results with other literature, we find general agreement that post-ACLR cartilage undergoes degenerative changes. Evidence linking compositional MRI biomarkers to patient-reported outcomes remains limited and inconsistent, with generally modest associations reported across knee osteoarthritis and other at-risk cohorts (61). While that review addressed reparative procedures, both contexts underscore T2 mapping as the most widespread and recognized compositional MRI technique for knee cartilage (62). Our review specifically in ACLR patients echoes that conclusion – T2 was the most commonly assessed parameter and consistently showed changes, supporting its validity as a biomarker of early degeneration. Furthermore, longitudinal cohort studies (e.g., from longitudinal whole-joint MRI cohorts (22) and the MOON nested cohort (51)] have reported that ACL-injured knees are at elevated risk of radiographic osteoarthritis within 10–15 years. Our MRI-based findings provide a mechanistic bridge: even before radiographic OA becomes evident, quantitative MRI reveals the cartilage matrix is deteriorating. This helps explain why, a decade after ACLR, many patients develop osteoarthritic changes. It also aligns with long-term follow-ups of ACL cohorts that show higher rates of OA compared to uninjured populations. In summary, our results reinforce the paradigm that ACLR is not a curative procedure with respect to cartilage health—rather, post-traumatic osteoarthritis appears to be set in motion at the time of injury and continues to evolve, with ACLR perhaps only partially mitigating the risk.

### Interpretation and strength of evidence

Interpretation was restricted to what can be supported directly by the included imaging studies. Evidence for differential cartilage outcomes by graft choice remains limited because graft type was inconsistently reported and only a small number of studies allowed meaningful comparison, with mixed findings across cohorts (7, 48, 59). Similarly, rehabilitation exposure was seldom quantified in a way that permits attribution of compositional change to a specific protocol. Where biomechanics or functional status were examined, associations were reported rather than causal effects (7, 32, 39, 44, 55). These constraints require conservative inference and emphasize the need for prospective studies that standardize both rehabilitation characterization and imaging outcomes.

It is also important to distinguish MRI biomarkers from direct biochemical measurement. Compositional MRI indices are interpreted as surrogates of matrix status, but none of the included studies concurrently measured synovial or serum inflammatory mediators, and mechanistic explanations should therefore be framed as biological plausibility rather than demonstrated pathways (11, 60). In this context, the consistency of T2 mapping abnormalities across studies supports its utility as a non-invasive marker of early cartilage matrix disruption, particularly when interpreted alongside established principles of cartilage imaging (62).

### Limitations of the evidence base

Several limitations of the included studies and our synthesis should be acknowledged. First, MRI protocol heterogeneity was substantial, including differences in field strength (1.5 T vs. 3 T), sequence implementations, cartilage segmentation approaches, and outcome definitions. These variations limit direct comparability and reduce the validity of pooling. Absolute relaxation times can differ by scanner and acquisition, and studies variably reported superficial and deep layer values vs. full thickness averages. We therefore emphasized within-study comparisons and used standardized metrics when pooling was feasible, but heterogeneity remained high in quantitative analyses, reducing confidence in any single pooled effect size and supporting interpretation based on direction and consistency rather than precise magnitude.

Second, non-independence of data is possible because several studies were produced by the same research groups and may reflect partially overlapping cohorts. Sensitivity checks that de-emphasized potentially overlapping datasets did not change the overall direction of findings, but overlap could inflate apparent consistency. Where overlap was suspected, we prioritized the most informative dataset per outcome in quantitative synthesis.

Third, risk of bias remains a concern. Many studies were small and underpowered for subgroup analyses, and control selection was sometimes imperfect, particularly when using the contralateral knee as a comparator. Reporting of blinding and prespecified hypotheses was inconsistent, and selective reporting of sequences or subregions cannot be excluded. Publication bias is also possible, as studies with significant findings may be more likely to be published.

### Limitations of our review process

This review has limitations at the review level. Although a comprehensive search was performed across multiple databases and the protocol was registered in PROSPERO, quantitative synthesis was feasible for only a subset of outcomes because reporting formats and regions of interest varied substantially across studies (28, 63). Formal assessment of publication bias was not appropriate because fewer than 10 studies contributed to any single pooled analysis, limiting the interpretability of funnel plots or small-study effect tests. The possibility of missed evidence also remains due to restriction to English language publications and incomplete reporting of study methods in some primary reports.

### Implications for clinical practice

From a clinical perspective, our findings carry cautionary implications: patients and clinicians should be aware that cartilage health may be compromised even after a “successful” ACL reconstruction. The presence of biochemical cartilage changes on MRI suggests an elevated risk of developing osteoarthritis in the longer term. Thus, secondary prevention strategies should be considered. These might include interventions such as meniscal preservation (whenever possible, repair meniscus rather than remove), neuromuscular rehabilitation to normalize gait and loading, and possibly disease-modifying agents (although none are proven yet for PTOA prevention). While it is premature to change clinical protocols based solely on imaging biomarkers, the consistent signal of cartilage matrix degeneration implies that monitoring ACLR patients for early OA signs is warranted. MRI-based compositional imaging could even serve as an endpoint in future clinical trials (e.g., trials of novel injections or orthotics to slow PTOA)—our review establishes benchmark data for what changes can be expected in untreated cases.

We refrain from making strong recommendations about rehabilitation intensity or graft choice based on current evidence. The trend that *greater quadriceps strength correlates with better cartilage MRI* is hypothesis-generating and reinforces that rehabilitation focusing on muscle strengthening might be beneficial not just for function but possibly for joint health. However, high-quality studies (e.g., randomized trials comparing rehab protocols with cartilage outcomes) are needed before any definitive guidelines can be formed.

### Future research directions

Future work should prioritize longer-term longitudinal cohorts (beyond 5–10 years) to define how compositional MRI changes evolve toward structural osteoarthritis and to clarify whether these biomarkers track clinically meaningful outcomes. Although some included studies reported associations between KOOS and T2 or T1*ρ* measures,43 the relationship between imaging biomarkers and symptoms remains inconsistent and requires better powered, standardized studies.

Interventional research is also needed to test whether cartilage protective strategies after ACL reconstruction can modify compositional MRI trajectories, particularly in high-risk patients with meniscal pathology. On the technical side, greater standardization of compositional MRI protocols would improve cross-study comparability and enable more reliable pooling. Advanced techniques may also add sensitivity for early deep-zone changes; for example, UTE-T2* mapping detected a high prevalence of subsurface cartilage matrix abnormalities approximately 2 years after ACL reconstruction (48). Finally, integrated designs combining imaging with biomechanical and biochemical measures are needed to move from association toward mechanism and to support targeted prevention of post-traumatic osteoarthritis.

## Conclusions

In summary, this systematic review provides robust evidence that ACL reconstruction is followed by adverse biochemical and structural changes in the knee articular cartilage, detectable by quantitative MRI. The Results indicate that even after mechanical stability is restored, the cartilage shows signs of matrix deterioration (loss of proteoglycans and collagen integrity), especially in the medial compartment. These changes are influenced by concomitant injuries (meniscal damage worsens them) and appear to evolve over time, aligning with the known risk of post-traumatic osteoarthritis. Our Discussion places these findings in context, acknowledging the limitations while underscoring the clinical relevance: there is a window of opportunity after ACLR where interventions to protect cartilage might be beneficial. The review also highlights the need for standardized imaging and further longitudinal research to ultimately improve joint outcomes for ACLR patients. In accordance with PRISMA guidelines and ICMJE recommendations, we have tried to report these findings clearly and objectively, avoiding overinterpretation. The evidence to date paints a cautious but clear picture: the molecular pathology of cartilage post-ACL injury persists despite reconstruction, warranting ongoing attention in both research and clinical management.

## Appendix A

### Search strategies for electronic databases

The following search strategies were used to identify relevant studies in each database. The search terms were adapted to the specific syntax and requirements of each database.

PubMed

(“anterior cruciate ligament reconstruction” OR “ACLR” OR “ACL surgery”) AND (“cartilage biochemistry” OR “T1ρ mapping” OR “T2 mapping” OR “GAG mapping” OR “proteoglycans” OR “collagen”) AND (“magnetic resonance imaging” OR “MRI” OR “quantitative MRI”).

2. Scopus

TITLE-ABS-KEY ((“anterior cruciate ligament reconstruction” OR “ACLR” OR “ACL surgery”)) AND TITLE-ABS-KEY ((“cartilage biochemistry” OR “T1ρ mapping” OR “T2 mapping” OR “GAG mapping” OR “proteoglycans” OR “collagen”)) AND TITLE-ABS-KEY ((“magnetic resonance imaging” OR “MRI” OR “quantitative MRI”)).

3. Web of Science

TS = ((“anterior cruciate ligament reconstruction” OR “ACLR” OR “ACL surgery”)) AND TS = ((“cartilage biochemistry” OR “T1ρ mapping” OR “T2 mapping” OR “GAG mapping” OR “proteoglycans” OR “collagen”)) AND TS = ((“magnetic resonance imaging” OR “MRI” OR “quantitative MRI”)).

4. Cochrane Library

(“anterior cruciate ligament reconstruction” OR “ACLR” OR “ACL surgery”) AND (“cartilage biochemistry” OR “T1ρ mapping” OR “T2 mapping” OR “GAG mapping” OR “proteoglycans” OR “collagen”) AND (“magnetic resonance imaging” OR “MRI” OR “quantitative MRI”).

5. Embase

(“anterior cruciate ligament reconstruction'/exp OR “ACLR'/exp OR ‘ACL surgery'/exp) AND (‘cartilage biochemistry'/exp OR ‘T1ρ mapping'/exp OR ‘T2 mapping'/exp OR ‘GAG mapping'/exp OR ‘proteoglycans”/exp OR ‘collagen'/exp) AND (‘magnetic resonance imaging'/exp OR ‘MRI'/exp OR ‘quantitative MRI'/exp).

6. Gray Literature (Google Scholar)

(“anterior cruciate ligament reconstruction” OR “ACLR” OR “ACL surgery”) AND (“cartilage biochemistry” OR “T1ρ mapping” OR “T2 mapping” OR “GAG mapping” OR “proteoglycans” OR “collagen”) AND (“magnetic resonance imaging” OR “MRI” OR “quantitative MRI”).

**Notes on Search Strategy**

Boolean Operators: The terms “AND” and “OR” were used to combine search terms and ensure a comprehensive search.
Truncation and Wildcards: Truncation (*) and wildcards (?) were used where applicable to capture variations in spelling and word endings* (*e.g., “reconstruct*” for “reconstruction” or “reconstructing”).
Filters: No date or language filters were applied during the initial search to ensure no relevant studies were excluded.
Manual Screening: GAG-related search terms (for example, “GAG mapping” and dGEMRIC-related terminology) were included to maximize retrieval of compositional cartilage MRI literature. However, no eligible study in the final included set reported dGEMRIC or other direct GAG sensitive indices in a form suitable for synthesis.
